# Non-invasive Fecal Steroid Measurements for Monitoring the Reproductive Status of a Critically Endangered Yangtze Finless Porpoises (*Neophocaena asiaeorientalis asiaeorientalis*)

**DOI:** 10.3389/fendo.2019.00606

**Published:** 2019-09-04

**Authors:** Yu-jiang Hao, Ghulam Nabi, Xiao-Jun Deng, Ding Wang

**Affiliations:** ^1^Institute of Hydrobiology, Chinese Academy of Sciences, Wuhan, China; ^2^University of Chinese Academy of Sciences, Beijing, China; ^3^Key Laboratory of Animal Physiology, Biochemistry and Molecular Biology, College of Life Sciences, Hebei Normal University, Shijiazhuang, China

**Keywords:** Yangtze finless porpoise, endangered, endocrinology, conservation, reproduction

## Abstract

Yangtze finless porpoise (*Neophocaena asiaeorientalis asiaeorientalis*) is a critically endangered freshwater cetacean dwelling in the Yangtze River and its adjoining lakes. Affected mainly by the various anthropogenic activities in this region, its population decreased dramatically in the past two decades. To protect this animal from extinction, captive breeding program is an important way to provide basic knowledge for wild population conservation. Non-invasive fecal steroid radioimmunoassay technique was validated in three captive Yangtze finless porpoises for the first time in this study. The seasonality of one captive male and the reproductive status of two females were investigated by longitudinal monitoring their fecal reproductive steroid hormone metabolites. Pregnancy could be diagnosed by an abrupt increase in fecal progesterone metabolites. In late pregnancy (4 months before birth), a significant decrease of fecal progesterone metabolites was observed, which might be referenced for the expectation of parturition date. Seven estrous cycles were recognized in one breeding season of a non-pregnant female judged by the variation of fecal progesterone metabolite levels. The fecal progesterone metabolite level was proved a reliable and precise indicator for estrus and pregnancy diagnosis.

## Introduction

The Yangtze finless porpoise (*Neophocaena asiaeorientalis asiaeorientalis*) is a critically endangered (1) freshwater subspecies of *Neophocaena*, which lives only in the middle and lower reaches of the Yangtze River and its appended big lakes (2). With the increasing anthropogenic impacts on this subspecies, its population dropped quickly in the past two decades. The population was estimated approximately, 1800 in 2006 (3), which was only one-third of that reported in the 1990s (4). However, the survey conducted in December 2012 revealed that the population status is even getting worse (2). Moreover, population viability analysis suggests that this subspecies may follow the fate of extinct baiji (*Lipotes vexillifer*) (5), within the next 15 years if no practical conservation efforts could be taken promptly (6).

In order to protect this animal from extinction like the baiji, and preserve the genetic diversity in captivity, a captive breeding program has been launched since the early 1990s by the Institute of Hydrobiology of the Chinese Academy of Sciences. A small captive population has been successfully established since 1996. Three calves have been born in this captive group since 2005, which presents the first reproductive success of freshwater cetacean in captivity (7). Moreover, this program provides a unique opportunity to promote our understandings on biology, particularly reproductive physiology of this critically endangered cetacean. By monthly monitoring of the serum hormones, Chen et al. (8) found that Yangtze finless porpoises (YFPs) are obvious seasonal breeders. The serum testosterone concentrations of the male finless porpoise increased from March, peaked in April through July, dropped in September, and kept quiescent in the rest of the months. However, probably because of the possible effects of the stress from monthly manipulation on reproductive hormone secretion (9–11), none of the female animals had ever been pregnant in those consecutive study years. Moreover, it is difficult to monitor the ovarian cycles and hormone dynamics in females through this monthly blood sampling regime because of the complicated variations of the reproductive hormones in females.

Unfortunately, we still know little about the ovarian cycles and hormone dynamics in ovulation of the female YFPs so far due to the restriction of methodology. Our present limited knowledge of the basic reproductive physiology of the female YFP was all achieved from the wild ecological observations and anatomic works, and the conclusions are quite controversial (12). Chen et al. (13) firstly concluded that the gestation length of the YFP was about 10 months by calculating through the body length of the newborn calves. However, using similar calculations, other studies concluded that the gestation length of this animal was only about 9.4 months (14), or 10.5 months (15). These results easily gave a false impression that the YFPs may have a shorter gestation length compared with other small toothed cetaceans since most of the other small toothed cetaceans have a relatively longer pregnancy period (less or longer than 12 months) (16). However, worrying about the further impacts of capture stress on the reproduction of the animals, we are cautious to increase blood-sampling frequency of the animals. Therefore, it seems indispensable to seek an alternative non-invasive technique to further understand the reproductive physiology of this endangered cetacean.

Techniques for non-invasive analysis of fecal or urinary steroid metabolites have been quickly developed in the past 30 years. These techniques have been used to assess reproductive status and function in a wide range of terrestrial animals both in captive and wild studies (17–20). The techniques now are widely accepted in many studies in which blood sampling on a regular basis was either difficult or impossible (21). Inspiringly, this technique has also been successfully used in some cetacean species. Rosalind et al. (22) describes the validation of radioimmunoassay techniques to study reproduction in right whales (*Eubalaena glacialis*) by measuring fecal metabolites, which may present the first application of this methodology in wild cetaceans. Their work shows that this technique could be used to determine gender, detect pregnancy and lactation, and assess age at sexual maturity in right whales. They also proposed its potential for use in other endangered cetacean populations. Robeck et al. (23–25) developed and applied the non-invasive technique in well-trained captive cetacean species (bottlenose dolphins (*Tursiops truncatus*), killer whales (*Orcinus orca*), and Pacific white-sided dolphins (*Lagenorhynchus obliquidens*) by monitoring the excretory dynamics of urinary Luteinizing Hormone (LH) and reproductive steroid metabolites. Not only the basic reproductive physiology of the cetaceans could be clearly described, but, ovulation and pregnancy could also be precisely detected and diagnosed by determining the dynamics of LH and ovary steroid metabolites in their studies. This technique vastly improved the development of artificial insemination in aquatic animals.

Encouraged by these achievements in cetacean non-invasive reproduction research, the present study was conducted to (1) validate the non-invasive steroid metabolite monitoring technique to be used in the captive YFP for reproductive research; (2) to determine the excretory dynamics of the reproductive steroids, particularly the dynamics of the ovarian steroid metabolites of the female porpoises; (3) to understand the basic reproductive physiology, such as ovulation, pregnancy, and birth etc. of the YFPs and provide information for wild population conservation.

## Materials and Methods

### Animals

Three YFPs comprising one male (10 years) and two females (7 and 10 years, respectively) were involved in this study. The male (A-Fu) and the elder female porpoise (Ying-Ying) were introduced into the Baiji Dolphinarium in November 1996, and the young female (Jing-Jing) was introduced in November 1999. The animals were housed in a kidney-shaped rearing pool (20 × 7 × 3.5 m) in the rearing hall of the aquarium. During the study period, Jing-Jing was pregnant. The male was therefore separated into an adjacent round pool to avoid the sexual disturbance from the male to the pregnant female. The round pool is connected with the kidney-shaped pool through a channel, which was blocked with a stainless-steel fence. The animals in both sides could “see” each other visually and acoustically through the fence but could not contact physically. The YFPs were mainly fed with crucian carps (*Carassius auratus*), supplemented with some small common carps (*Cyprinus carpio*) at ~6–8% of their body weight per day. The water quality is monitored daily to keep it in the optimum conditions through a connected filtrating-disinfecting system. The water temperature changed seasonally ranging from 13 to 27°C.

### Training and Sampling

**Figure 1:**
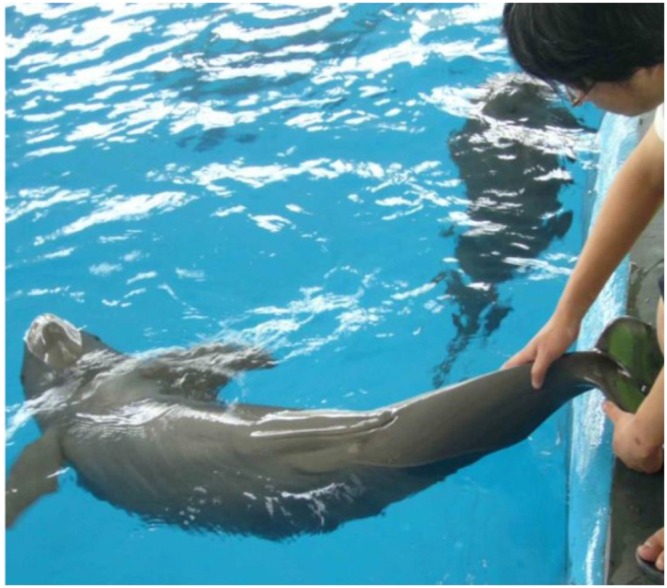
Fecal and urine collection from the YFP.

The animals were trained with positive reinforcement technique following the rules described by Pryor (26). Briefly, the animals were instructed to turn their body over with the behavioral command of the trainers. Then the trainers gently dab the genital area of the animals with the fingers (Figure 1). If the animals defecate naturally, the samples were collected with a spoon and transferred into a plastic tube with a cap. The samples were labeled and immediately stored at −20°C until assay. All procedures described within this work were reviewed and approved by the veterinarian of the Baiji Dolphinarium and were conducted in accordance with the local Regulations for the Administration of Experimental Animals.

The fecal samples of male porpoise were collected during the study period (from November 2004 to February 2006), however, only the samples collected about each 10 days were selected for steroid metabolite assay. The pregnant female, Jing-Jing, gave birth to a male porpoise on July 5th, 2005. The porpoise began to accept fecal sample collection in December 2004, and her pregnancy was estimated at the end of July or beginning of August in 2004. Therefore, this study only presented the data of middle and late stages of her pregnancy. The porpoise became fretted and kept distance from the trainer a week before parturition. The last fecal sample before birth was collected on June 29th. The first fecal sample after parturition was collected a month later on August 8th. Four blood samples, including three in pregnancy and one in lactation were collected during the study period. The fecal samples of non-pregnant adult porpoise were collected from April 2005 to February 2006 (333 days in total). The samples collected after every 5 days were analyzed for steroid hormones. Ethical approval (31430080) for the study was obtained from the Research Ethic Committee of Institute of Hydrobiology, University of the Chinese Academy of Sciences, Wuhan, China. The study strictly adhered to the Chinese Law and ethical guidelines for biodiversity.

### Fecal Steroid Extraction and Radioimmunoassays (RIA)

Feces were extracted with methanol following the method described by previous works (27–29) with some modifications. Briefly, the frozen fecal samples were lyophilized at −45°C, pulverized, and sieved through a fine mesh to remove the undigested scales and fish bones. Precisely weighed fecal powder (about 0.1 g) were mixed with 1 mL methanol, vortexed for 1 min, and then extracted for 30 min in a horizontal shaker at 25°C. Extracts were centrifuged at 3,000 rpm at normal temperature for 20 min and the supernatant was transferred into a new plastic tube. The pellet was resuspended in 0.5 mL methanol and vortexed for 1 min and then recentrifuged for 20 min. The supernatant was collected and pooled with the first extract in the tube. The pooled supernatant was heated by circulating water bath until methanol was totally evaporated. The dried extracts were re-dissolved in 400 μL 0.05 M PBS-BSA (0.1% bovine serum albumin, vortexed for 1 min, then sonicated for 30 s. All these operations were conducted in a fume hood. The efficiencies of extraction process were determined by monitoring the recovery of [^125−^I] progesterone (2,000 cpm), [^125−^I], estrogen (2,000 cpm), and [^125−^I] testosterone (3,000 cpm) added to each subset of samples (*n* = 10) prior to extraction. The extraction efficiencies for progesterone, estrogen and testosterone were 86.6 ± 4.3, 60.9 ± 5.5, and 86.0 ± 3.8%, respectively.

The steroids in the diluted extracts were assayed using the commercial magnetic microparticle radioimmunoassay kits provided by Beijing Chemclin Co., China. All the assays were performed according to the instructions of the kits. The assay procedures for the steroid hormones were quite similar. Briefly, taking the progesterone assay, for example, 100 μL progesterone standard (or analyte), 100 μL ^125^I-lablled progesterone, and 100 μL anti-serum were orderly added into the reaction tube (12 × 60 mm) and oscillated for 30 min at 37°C. Then 500 μL magnetic separation reagent was added into the tube and the tube was kept still for 10 min at room temperature. Then the reaction tube was placed onto the magnetic board for 10 min for separation. The liquid section was dumped, and the radioactive counts were measured by an automatic analyzer (30 s). The standard of this kit ranged from 0 to 100 ng/mL. The sensitivity of this assay was 0.05 ng/mL. The variation coefficients for intra and inter-assay were 7.0 and 10.0%, respectively. The assay procedures for estrogen and testosterone were quite similar to that for progesterone assay except for the volume of the analytes (50 μL). The standards of testosterone and estrogen ranged from 0 to 2,000 ng/mL. The sensitivity of the testosterone assay was 0.2 ng/mL. The variation coefficients of intra and inter-assay were 7.4 and 9.5%, respectively. The sensitivity of estrogen assay was 0.5 ng/mL. The variation coefficients of intra- and inter-assay were 6.0 and 7.7%, respectively. In all assays, standard curves were used to determine the concentration of steroid hormone metabolites in the diluted extracts. The final concentrations of the hormone metabolites in fecal samples, expressed as ng/g dry weight was calculated according to the dilution ratio and exact weight of the samples.

### Laboratory and Biological Validation

**Figure 2:**
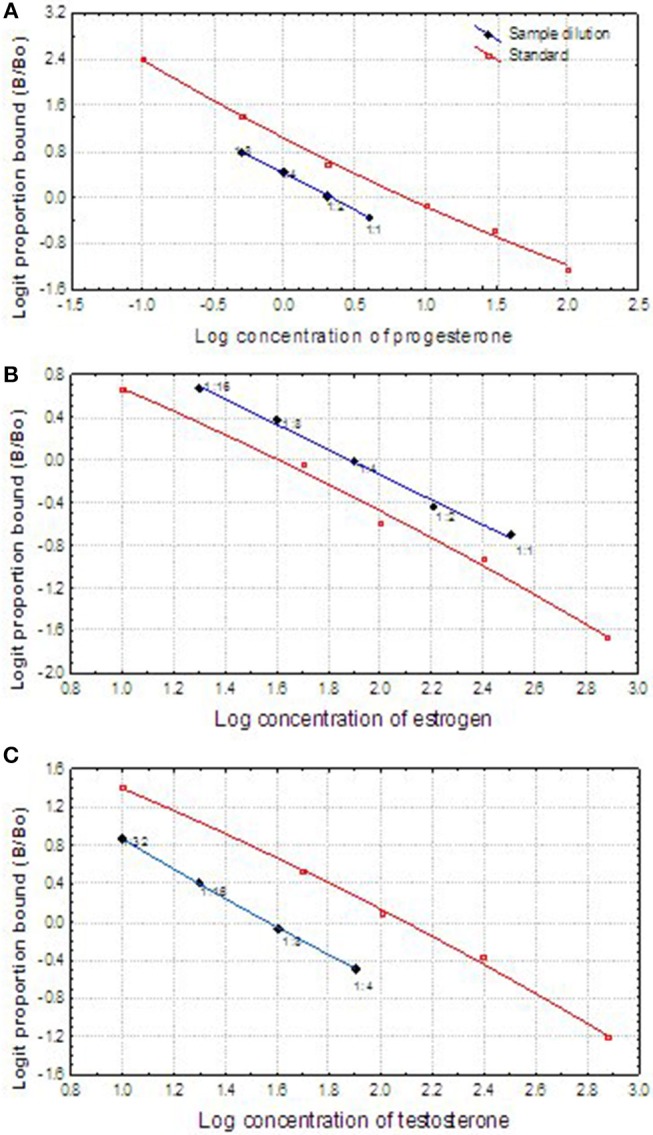
Displacement comparison of log-logit transformed curves of fecal reproductive steroids (progesterone, estradiol, and testosterone) serial dilutions, and the log-logit curves of the steroid standards showing the displacement of the curves.

The fecal steroid assay was validated by (1) running parallelism between the serially 2-fold diluted extracts of pooled fecal samples and the respective standard curves; (2) comparing the steroid metabolite concentrations of the pooled fecal samples collected during the known reproductive status of male or female animals. The logit transformed sample curves of progesterone, estrogen and testosterone all paralleled the respective logit transformed standard curves, respectively (Figures 2A–C), which demonstrated that the cross-reacted hormone metabolites in the fecal samples were immunologically similar to the standards and could be measured proportionately. The appropriate dilution ratios for each assay were determined based on the binding inhibition observed at ~50% (or 0 in the logit figures). Consequently, the re-dissolved extracts were diluted in proper proportion (1:12 for testosterone in the breeding season and 1:6 in non-breeding seasons; 1:4 for progesterone, and 1:12 in pregnant females) in the PBS-BSA buffer before running the appropriate RIA. Estrogen both in male and female samples could be assayed directly without dilution except only a few samples collected in the breeding season from the male.

Physiological validation was further performed to determine if the hormonal measures accurately reflect the physiological events of interest. The YFPs are obvious seasonal breeders and the reproductive status of mature males has been demonstrated by the intensity of their sexual behavior (30) and serum testosterone concentrations (8). Pregnancy of female porpoise could be diagnosed by B-mode ultrasound. Ten samples from a pregnant female, and non-pregnant female were randomly selected for progesterone and estrogen comparisons. Ten samples collected from a mature male in breeding and non-breeding season were used for testosterone comparison (Table 1). The progesterone concentrations in the fecal samples of the pregnant female were nearly 10 times higher than those in the non-pregnant female, and the fecal testosterone concentration of the male porpoise in a breeding season was also much higher than that in the non-breeding season, which demonstrated that the fluctuations of fecal steroid metabolites physiologically reflected the relevant events.

**Table 1:** The physiological validation of the fecal reproductive steroids (*t*-test).

**Fecal steroids**	**Pregnant**	**Non-pregnant female**	**Male in breeding season**
Progesterone (ng/g)	1,524.1 ± 1,047.7^a^ (*n* = 10)	151.9 ± 152.2^b^ (*n* = 10)	–
Testosterone (ng/g)	–	1,232.0 ± 787.9^a^ (*n* = 10)	20,060.8 ± 12,029.9^b^ (*n* = 10)
Estradiol (pg/g)	333.2 ± 112.5^a^ (*n* = 10)	1,040.4 ± 639.2^b^ (*n* = 10)	667.8 ± 375.6^a, b^ (*n* = 10)

*n, sample size; significant difference (P < 0.05) between the values were demonstrated with different superscripts a and b*.

### Blood Sampling and Analysis

Detail of the animal handling, sample collection, and already validated assays are explained in detail by Hao et al. (31) and Nabi et al. (32, 33).

### Statistical Analysis

All the data were analyzed by the Statistica software for windows. The independent sampling *t-*test was used for two group comparisons. ANOVA was used for comparison of the steroid concentrations among the different reproductive status. All the data for comparison were presented as Mean ± SD. The variations of the various hormones were simply presented by Scatterplot provided by the Statistica software. A *P* < 0.05 indicated a statistically significant difference.

## Results

### Fecal Steroid Metabolites in the Mature Male

**Figure 3:**
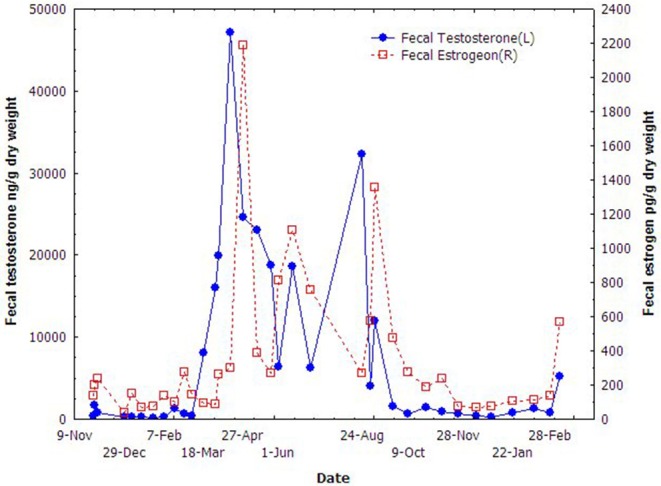
Seasonal variations of the fecal testosterone and estradiol of the male YFP.

The results of fecal testosterone and estrogen metabolites of the male porpoise are shown in Figure 3. The concentrations of both fecal steroid metabolites varied seasonally and demonstrated quite similar annual trends. They increased promptly in March, dropped quickly from September to October, and kept in very low level to the next February. Even though the two steroids remained in relatively higher-levels during March to September, both of them fluctuated significantly in the middle of this period during June–July.

### Fecal Steroids in Pregnant Females

**Figure 4:**
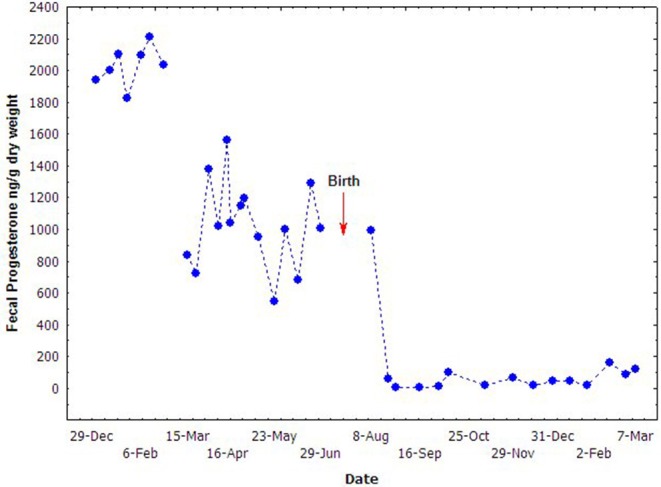
Fecal progesterone concentrations during different stages of pregnancy.

The concentration of fecal progesterone metabolites varied significantly during the middle and late stages of pregnancy (Figure 4). It seemed that the mid-March was a watershed. The concentrations of fecal progesterone metabolites kept in relatively higher level before mid March (9th, March), but dropped promptly since then and kept in relatively lower level until birth. The high concentration of fecal progesterone lasted another month after parturition before dropping to the normal level. The concentrations of the fecal steroid metabolites in different reproductive stages of the female were statistically compared and presented in Table 2. The concentration of fecal progesterone metabolites in the mid-pregnancy was much higher than that in later pregnancy and lactation period (*P* < 0.05). However, there was no discernible difference of fecal estrogen metabolites found in the three stages of the pregnant female porpoise (*P* > 0.05).

**Table 2:** The comparison the fecal steroids of the pregnant female porpoise in different stages of pregnancy and lactation period in captivity.

**Fecal hormones**	^*^Middle pregnancy	**Late pregnancy**	**Lactation period**	
Progesterone (ng/g)	2,030.2 ± 125.5^a^ (*n* = 7)	1,026.8 ± 278.3^b^ (*n* = 14)	55.6 ± 48.1^c^ (*n* = 14)	
Estradiol (pg/g)	330.1 ± 130.6 (*n* = 7)	335.7 ± 74.6 (*n* = 14)	360.4 ± 218.7 (*n* = 15)	
Testosterone (ng/g)	382.3 ± 134.3^a^ (*n* = 7)	1,306.1 ± 725.0^b^ (*n* = 14)	2,367.0 ± 521.5^b^ (*n* = 4)	492.1 ± 221.1^a^^**^ (*n* = 11)

*n, sample size; significant difference (P < 0.05) between the values were demonstrated with different superscripts a, b, and c*.

* *Middle and later pregnancy was divided based on the significant drop of progesterone concentration in middle March*.

** *The fecal testosterone after parturition were analyzed separately pre- and post-middle September (16th September) due to its significant concentration change in the fecal samples*.

**Figure 5:**
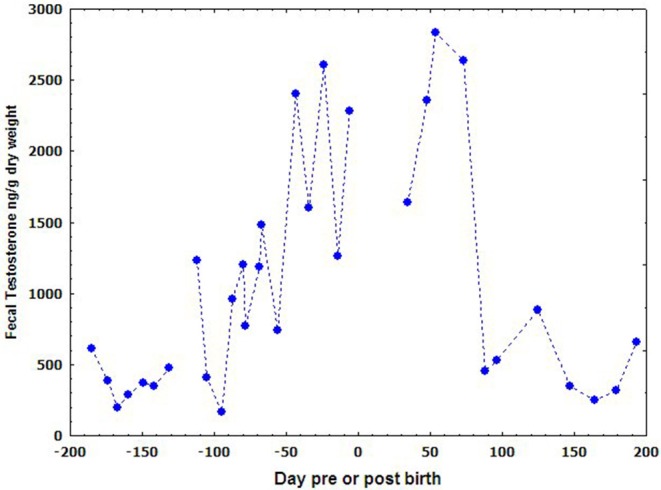
The variation of fecal testosterone concentration in pregnant YFP during pregnancy.

The fecal testosterone in the pregnant female was also assayed (Figure 5). Interestingly, the concentration of fecal testosterone metabolites increased obviously with the time of pregnancy and reached to its peak just before the birth on June 29th of 2005. It still kept in a much higher level in the first two and half months after parturition before returning to its normal level in *P* < 0.05, Table 2.

The concentrations of progesterone, estrogen, and testosterone in the blood samples were assayed and presented in Table 3. The serum concentrations of these steroids in different stages showed consistent profile with that in fecal samples.

**Table 3:** The serum progesterone, estradiol, and testosterone concentration of the pregnant female Yangtze finless porpoise during her pregnancy and lactation period in captivity.

**Date of sampling**	**Aug-30, 04**	**Nov-9, 04**	**Mar-14, 05**	**Oct-12, 05**
Progesterone (ng/mL)	65.8	66.4	22.9	0.3
Estradiol (pg/mL)	33.6	58.8	66.3	32.3
Testosterone (ng/mL)	0.5	9	49.5	–

### Fecal Steroids in Non-pregnant Mature Female

**Figure 6:**
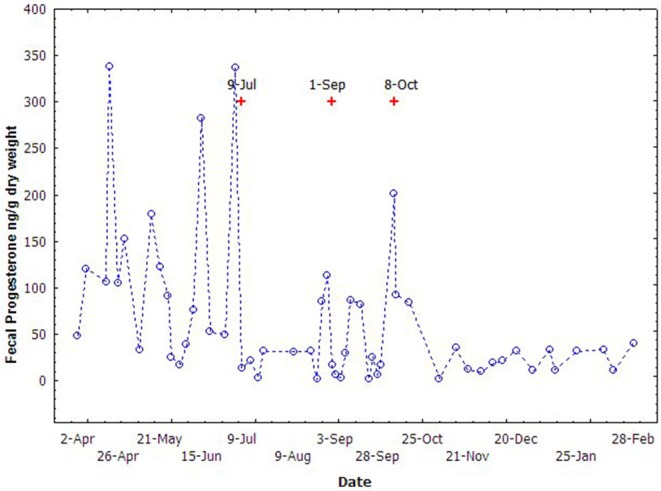
The cyclic variation of fecal progesterone concentrations of the non-pregnant female during breeding season (Apr to Nov) in 2005. The “+” indicate the time of vaginal mucus collection.

Variation in the concentrations of fecal progesterone metabolites of the non-pregnant female is presented in Figure 6. The fecal progesterone metabolites changed cyclically during April through October and kept quiescently in other months. A total of seven cycles of progesterone metabolites were recognized totally during this season. The fecal estrogen metabolites changed similarly with the profile of the fecal progesterone metabolites but, the cycles in estrogen were not as easily recognized as in the progesterone. The average length of a cycle was about 23.2 ± 4.5 days.

Vaginal mucus secreted during the cycles was also observed opportunistically by trainers during fecal sample collection. Since the vaginal mucus was not intentionally collected, only 3 events of vaginal mucus secretion were recorded. The time of the vaginal mucus secretion was consistent with the increase of the fecal progesterone metabolites, and generally occurred just 2–3 days after the peak of the progesterone metabolites (Figure 6).

## Discussion

### Validation for Fecal Steroid Assays

The main route of steroid metabolite excretion (urine or feces) can vary considerably among species as well as between steroids within the same species (17, 34). The biliary system excretes steroid metabolites into the duodenum and transport with the digesta. They are further metabolized during transportation through by residing bacteria, deconjugated, and excreted in the feces or re-absorbed into enterohepatic circulation and finally excreted through urine (18, 34). Therefore, it is essential to biologically validate assays and certify the biological meaningfulness of analyses (35). Like Florida manatee (*Trichechus manatus latirostris*) fecal samples, our physiological validation suggests significantly higher testosterone concentrations in males compared to non-pregnant adult females (36). Similarly, a significantly higher progesterone level in pregnant and estrogen in non-pregnant female samples also indicate fecal steroid validation as observed by Biancani (37) and Valenzuela-Molina et al. (38) in the feces of bottlenose dolphin and blue whales (*Balaenoptera musculus*). However, to further improve the validity for steroid hormone metabolites in feces, high-pressure liquid chromatography analysis is suggested. Further studies to relate fecal estrogen, progesterone and testosterone with sexual maturity will improve our understanding about the reproductive biology of YFPs.

### Seasonal Changes of Male Reproductive Hormones

Our results of reproductive seasonality in the fecal samples of mature male YFPs are parallel with the findings of Chen et al. (8) who observed reproductive seasonality in the serum samples of YFPs. Like our results, Chen et al. (8) reported increased serum testosterone concentrations in the male YFPs from March, dropped in September, and kept quiescent in the rest of months. As testosterone, a similar seasonal variation pattern in estrogen metabolites was also noted by Chen et al. (8). Both the studies confirm reproductive seasonality in YFPs which may begin as early as March and end as late as September however, this is the first study reporting seasonal variations through fecal samples. In spite of hormone analysis, March as a start of the breeding season was also observed on the basis of alteration in the sexual behavioral of male YFPs (30, 39). Like YFP, reproduction in most marine mammals is highly seasonal (40). All seals except, *Neophoca cinerea* are a seasonal breeder (41) with highly seasonal variation in testosterone level (42). A similar pattern of testosterone seasonality was found in the bottlenose dolphin. Serum testosterone levels start to elevate in April and peaked in July, with the lowest level found during September to November (43). Furthermore, in female cetacean, an increase in estrogen level is related with increase in the spermatogenesis, male density (43), and the positive effect induce by the dominant subjects (44). Unfortunately, in YFPs, no such data is available regarding seasonal variation in the sperm density which can affect the sexual behavior of female during breeding activities.

### Estrous Cycles of Non-pregnant Adult Females

In YFPs, we observed distinct variation in both fecal estrogen and progesterone with seven distinct cycles in progesterone. In bottlenose dolphins and sea lions (*Zalophus californianus*), variation in serum and plasma estrogen were also observed, but unlike YFPs, they peaked in September and decreased during April and May, respectively (45, 46). However, the secretions of estrogen from April to October needs further studies to investigate if folliculogenesis is not arrested (46). In YFPs, this could also possibly support the assumption that most of the cetaceans are seasonal breeders and spontaneous ovulators (47). Unlike YFPs, in bottlenose dolphin, seasonal serum and plasma progesterone concentrations peaked in June and declined during July and September (46), while in sea lion increased during September–November (45) suggesting species differences. In humpback dolphin (*Sousa chinensis)*, ovulation and conception may occur during the winter months and the individual differences found in the ovarian cycles of these animals needs further research to identify both extrinsic and intrinsic influencing factors (48) as both species and age can compromise the seasonal variation of hormone levels (47, 49). In Killer Whales, the secretion of a heavy percentage of vaginal mucous is often linked with receptivity or estrus. Though during various phases of reproductive cycles, mild vaginal mucous secretion occurs, but heavy secretion during a higher reproductive hormones levels are the result of altering sexual behavior and secretory changes which are essential for conception (50). In northern fur seal (*Callorhinus ursinus*), ovulation is indicated by a rise in the serum progesterone (51). Even once ovulation occurs, thecal cell and granulosa are transformed into progesterone-secreting small and large luteal cell (52) having different secretory capacities. Therefore, in YFPs, the link between rising in fecal progesterone metabolites and vaginal secretions might be the receptive behavior of females for conception.

### Reproductive Hormone Changes in Different Pregnancy Stages

In YFPs, during pregnancy, we observed a decreasing pattern of progesterone level from mid-pregnancy till birth. The decreasing progesterone level in later pregnancy might be the result of reducing the numbers of corpora lutea, as observed in the later stage of pregnant female African elephants (*Loxodonta africana*) with low progesterone compared to the highest progesterone level in mid-pregnancy (53). A similar increase in the progesterone level at mid pregnancy was also observed in the rat (54), *Sceloporus cyanogenys* (55), *Tiliqua rugosa* (56), and *Thamnophis elegans* (57), suggesting the possibility that a certain critical mass of luteal tissue may be required to maintain plasma levels of progesterone at a level compatible with pregnancy (58). Serum estrogen increased with advancing gestational age reaching a peak level close to the term in killer whales, baboons and humans, but in contrast to rhesus monkey where the estrogen level plateau during the last one-third of pregnancy (59, 60). However, in bottlenose dolphin, estrone conjugate (E1-C) was significantly higher during late compared to early and mid, while E3-C was significantly higher in early than mid and late pregnancy stages (61). Increased in the level of estrogen near the term is associated with the onset of parturition and to prepare the mammary gland for lactation (62). However, in YFPs, we did not find an increasing or decreasing pattern in the level of estrogen during the course of pregnancy. Further studies are needed to investigate various estrone conjugates and their role during pregnancy stages.

The testosterone level in YFPs carrying a male fetus increased significantly with advancing pregnancy and reached its peak level just before birth. As soon as fetal testes differentiate, they become steriodogenically active and start testosterone production (63), elevating maternal serum testosterone throughout gestation (64). To understand, whether, testosterone in YFPs can be used as a marker for gender discrimination requires additional studies with large sample size carrying both male and female fetuses. For example, in Asian elephants (*Elephas maximus*) (65), baboons (66), and lemurs (67) maternal serum testosterone can be used to determine the gender of fetus during gestation however, in bottlenose dolphin (61), human (68), marmoset (69), and rat (70) no such association is reported.

## Conclusions

Present study validated fecal steroid hormones in the critically endangered YFPs for the first time. Using a non-invasive fecal steroid radioimmunoassay technique, we observed seven estrous cycles in the non-pregnant adult female in one breeding season. Similarly, seasonality in male steroid hormones and use of the fecal progesterone metabolite for estrus and pregnancy diagnosis was reported. The use of non-invasive fecal steroid hormone analysis can help us to track, improve basic aspects, and provide species specific information of the reproductive endocrine activities. This will improve the breeding rates and help in creating self-sustaining population. Further studies are needed to correlate fecal steroid hormones with ultrasonography to monitor reproductive activities.

## Data Availability

The datasets generated for this study are available on request to the corresponding author.

## Author Contributions

YH conceived, collected, and analyzed the data. YH and GN drafted the manuscript. X-JD reviewed the manuscript. DW supervised the study.

### Conflict of Interest Statement

The authors declare that the research was conducted in the absence of any commercial or financial relationships that could be construed as a potential conflict of interest.

## References

[1] Wang D Turvey ST Zhao X Mei Z Neophocaena asiaeorientalis ssp. asiaeorientalis. The IUCN Red List of Threatened Species. Version 3.1. (2013). Available online at: http://www.iucnredlist.org/details/summary/43205774/0 (accessed May 8, 2018).

[2] Mei Z Zhang X Huang SL Zhao X Hao Y Zhang L The Yangtze finless porpoise: on an accelerating path to extinction? Biol Conserv. (2014) 172:117–23. 10.1016/j.biocon.2014.02.033

[3] Zhao X Barlow J Taylor BL Pitman RL Wang K Wei Z Abundance and conservation status of the Yangtze finless porpoise in the Yangtze River, China. Biol Conserv. (2008) 141:3006–18. 10.1016/j.biocon.2008.09.005

[4] Zhang X Liu R Zhao Q Zhang G Wei Z Wang X The population of finless porpoise in the middle and lower reaches of Yangtze River. Acta Theriol Sin. (1993) 13:260–70.

[5] Turvey ST Pitman RL Taylor BL Barlow J Akamatsu T Barrett LA. First human-caused extinction of a cetacean species? Biol Lett. (2007) 3:537–40. 10.1098/rsbl.2007.0292 17686754 PMC2391192

[6] Mei Z Huang SL Hao Y Turvey ST Gong W Wang D Accelerating population decline of Yangtze finless porpoise (*Neophocaena asiaeorientalis asiaeorientalis*). Biol Conserv. (2012) 153:192–200. 10.1016/j.biocon.2012.04.029

[7] Wang D Hao Y Wang K Zhao Q Chen D Wei Z. Aquatic resource conservation: the first Yangtze finless porpoise successfully born in captivity. Environ Sci Pollut Res Int. (2005) 12:247–50. 10.1065/espr2005.08.284 16206715

[8] Chen D Hao Y Zhao Q Wang D Reproductive seasonality and maturity of male *Neophocaena phocaenoides asiaeorientalis* in captivity: a case study based on the hormone evidence. Mar Freshw Behav Physiol. (2006) 39:163–73. 10.1080/10236240600563396

[9] Young KM Brown JL Goodrowe KL Characterization of reproductive cycles and adrenal activity in the black-footed ferret (*Mustela nigripes*) by fecal hormone analysis. Zoo Biol. (2001) 20:517–36. 10.1002/zoo.10001

[10] Young KM Walker SL Lanthier C Waddell WT Monfort SL Brown JL. Noninvasive monitoring of adrenocortical activity in carnivores by fecal glucocorticoid analyses. Gen Comp Endocrinol. (2004) 137:148–65. 10.1016/j.ygcen.2004.02.016 15158127

[11] Hao YJ Zhao QZ Wu HP Chen DQ Gong C Li L Physiological responses to capture and handling of free-ranging male Yangtze finless porpoises (*Neophocaena phocaenoides asiaeorientalis*). Mar Freshw Behav Phy. (2009) 42:315–27. 10.1080/10236240903302161

[12] Hao YJ Wang D Zhang XF Review on breeding biology of Yangtze finless porpoise (*Neophocaena phocaenoides asiaeorientalis*). Acta Theriol Sin. (2006) 26:191–200.

[13] Chen PX Renjun L Harrison J Reproduction and reproductive organs in *Neophocaena asiaeorientalis* from the Yangtze River. Aquat Mamm. (1982) 9:9–16.

[14] Jiang XF Study on the morphogenesis of foetus and reproductive regularity the finless porpoise, *Neophocaena phocaenoides*. Acta Hydrob Sin. (1995) 19:211–5.

[15] Chang Q Zhou KY The growth and reproduction of finless porpoise, *Neophocaena phocaenoides*, in the Yangtze River and Yellow/Bohai Sea. J Nan Norm Univ. (1995)18:114–24.

[16] Boyd IL Lockyer C Marsh HD Reproduction in marine mammals. In: Reynolds JE III Rommel SA, editors. Biology of Marine Mammals. Washington, DC; London: Smithsonian Institution Press (1999). p. 218–86.

[17] Schwarzenberger F Möstl E Palme R Bamberg E Faecal steroid analysis for non-invasive monitoring of reproductive status in farm, wild and zoo animals. Anim Reprod Sci. (1996) 42:515–26. 10.1016/0378-4320(96)01561-8

[18] Graham LH Non-invasive monitoring of reproduction in zoo and wildlife species. Annu Rev Biomed Sci. (2004) 6:91–8. 10.5016/1806-8774.2004v6p91

[19] Shimizu K. Studies on reproductive endocrinology in non-human primates: application of non-invasive methods. J Reprod Dev. (2005) 51:1–13. 10.1262/jrd.51.1 15750292

[20] Brown JL. Comparative endocrinology of domestic and nondomestic felids. Theriogenology. (2006) 66:25–36. 10.1016/j.theriogenology.2006.03.011 16620933

[21] Schwarzenberger F The many uses of non-invasive faecal steroid monitoring in zoo and wildlife species. Int Zoo Yearb. (2007) 41:52–74. 10.1111/j.1748-1090.2007.00017.x

[22] Rosalind RM Hunt KE Kraus SD Wasser SK Assessing reproductive status of right whales (*Eubalaena glacialis*) using fecal hormone metabolites. Gen Comp Endocrinol. (2005) 142:308–17. 10.1016/j.ygcen.2005.02.002 15935157

[23] Robeck TR Steinman KJ Gearhart S Reidarson TR McBain JF Monfort SL. Reproductive physiology and development of artificial insemination technology in Killer Whales (*Orcinus orca*). Biol Reprod. (2004) 71:650–60. 10.1095/biolreprod.104.027961 15115725

[24] Robeck TR Steinman KJ Yoshioka M Jensen E O'Brien JK Katsumata E. Estrous cycle characterisation and artificial insemination using frozen–thawed spermatozoa in the bottlenose dolphin (*Tursiops truncatus*). Reproduction. (2005) 129:659–74. 10.1530/rep.1.00516 15855629

[25] Robeck TR Steinman KJ Greenwell M Ramirez K Van BW Yoshioka M. Seasonality, estrous cycle characterization, estrus synchronization, semen cryopreservation, and artificial insemination in the Pacific white-sided dolphin (*Lagenorhynchus obliquidens*). Reproduction. (2009) 138:391–405. 10.1530/REP-08-0528 19494046

[26] Pryor K Don't Shoot the Dog: The New Art of Teaching and Training. New York, NY: Simon and Schuster, Bantam Publisher (1999).

[27] Waseer SK Risler L Robert AS Excreted steroids in primate feces over the menstrual cycle and pregnancy. Biol Reprod. (1988) 39:862–72. 10.1095/biolreprod39.4.862 3207809

[28] Schwarzenberger F Fredriksson G Schaller K Kolter L. Fecal steroid analysis for monitoring reproduction in the sun bear (*helarctos malayanus*). Theriogenology. (2004) 62:1677–92. 10.1016/j.theriogenology.2004.03.007 15511554

[29] Molinia F La Falci S Myers V McLane D Non-Invasive Monitoring of Stoat Reproductive Hormones. Wellington: Science for Conservation 276, Department of Conservation (2007). p. 24.

[30] Wei Z Wang D Zhang F Wang K Chen DQ Zhao QZ Observation on some sexual behavior of the Yangtze finless porpoise (*Neophocaena phocaenoides asiaerientals*) in captivity. Acta Theriol Sin. (2004) 24:98102.

[31] Hao YJ Chen DQ Zhao QZ Wang D. Serum concentrations of gonadotropins and steroid hormones of *Neophocaena phocaenoides asiaeorientalis* in middle and lower regions of the Yangtze River. Theriogenology. (2007) 67:673–80. 10.1016/j.theriogenology.2006.06.014 17196248

[32] Nabi G Hao Y Zeng X Wang D Assessment of Yangtze finless porpoises (*Neophocaena asiaorientalis*) through biochemical and hematological parameters. Zool Stud. (2017) 56:31 10.6620/ZS.2017.56-31 PMC6517755 31966230

[33] Nabi G Hao Y McLaughlin RW Wang D. The possible effects of high vessel traffic on the physiological parameters of the critically endangered Yangtze Finless Porpoise (*Neophocaena asiaeorientalis ssp*. *asiaeorientalis*). Front Physiol. (2018) 9:1665. 10.3389/fphys.2018.01665 30546317 PMC6280126

[34] Palme R Fischer P Schildorfer H Ismail MN Excretion of infused 14C-steroid hormones via faeces and urine in domestic livestock. Anim Reprod Sci. (1996) 43:43–63. 10.1016/0378-4320(95)01458-6

[35] Goymann W On the use of non-invasive hormone research in uncontrolled, natural environments: the problem with sex, diet, metabolic rate and the individual. Methods Ecol Evol. (2012) 3:757–65. 10.1111/j.2041-210X.2012.00203.x

[36] Larkin ILV Gross TS Reep RL Use of faecal testosterone concentrations to monitor male Florida manatee (*Trichechus manatus latirostris*) reproductive status. Aquat Mamm. (2005) 31:52–61. 10.1578/AM.31.1.2005.52

[37] Biancani B Use of faecal samples to monitor the oestrous cycle, reproductive status and adrenal gland activity in the bottlenose dolphin (Tursiops truncatus) (Dissertation). Department of Veterinary Experimental Sciences, Universita' Degli Studi di Padova, Padua, Italy (2008).

[38] Valenzuela-Molina M Atkinson S Mashburn K Gendron D Brownell RL Jr. Fecal steroid hormones reveal reproductive state in female blue whales sampled in the Gulf of California, Mexico. Gen Comp Endocrinol. (2018) 261:127–35. 10.1016/j.ygcen.2018.02.015 29476760

[39] Hua YY Xiang CS Dong ML Zhang XF Chen NG Xu XM Study on the sexual and feeding behavior of the captured black finless porpoise (*Neophocaena phocaenoides*) in Yangtze River. Res Environ Yangtze Vall. (1994) 3:140–6.

[40] Atkinsonm S Yoshioka M Endocrinology of reproduction. In: Jamieson BGM, editor. Reproductive biology and Phylogeny of Cetacea. Enfield, NH: Science Publisher (2007). p. 171–92. 10.1201/b11001-7

[41] Atkinson S Reproductive biology of Seal. Rev Reprod. (1997) 2:175–94. 10.1530/ror.0.0020175 9414481

[42] Atkinsonm S Gilmartin WG Seasonal testosterone pattern in Hawaiian monk seal (*Monachus schauinslandi*). J Reprod Fertil. (1992) 36:35–9. 10.1530/jrf.0.0960035 1432968

[43] Schroeder JP Keller KV. Seasonality of serum testosterone levels and sperm density in *Tursiops truncates*. J Exp Zool. (1989) 249:316–21. 10.1002/jez.1402490310 2708948

[44] Whitehead H Mann J Female reproductive strategies of cetaceans. In: Mann J Connor RC Tyack P Whitehead H, editors. Cetacean Societies: Field Studies of Dolphins and Whales. Chicago: University of Chicago Press (2000). p. 219–46.

[45] Denise JG Kendall LM Matthew R Frances MDG Terrie MW Shannon A Seasonal changes in circulating progesterone and estrogen concentrations in the california sea lion (*Zalophus californianus*). J Mammal. (2007) 88:67–72. 10.1644/06-MAMM-A-060R2.1

[46] Fragalà S Medica P Grande F Vazzana I Fazio E. Evaluation of seasonal changes of serum and plasma estradiol-17β, progesterone and testosterone in dolphins (*Tursiops truncatus*) by chemiluminescence. Vet World. (2015) 8:977–82. 10.14202/vetworld.2015.977-982 27047185 PMC4774764

[47] Pomeroy P. Reproductive cycles of marine mammals. Anim Reprod Sci. (2011) 124:184–93. 10.1016/j.anireprosci.2010.08.021 20869821

[48] Brook F Evan HTL Frederic HCC Bruce M Assessment of the reproductive cycle of the Indo-Pacific Humpback Dolphin, *Sousa chinensis*, using ultrasonography. Aquat Mamm. (2004) 30:137–48. 10.1578/AM.30.1.2004.137

[49] Atkinson S Combelles C Vincent D Nachtigall P Pawloski J Breese M. Monitoring of progesterone in captive female false killer whales *Pseudorca crassidens*. Gen Comp Endocrinol. (1999) 115:323–32. 10.1006/gcen.1999.7319 10480983

[50] Robeck TR Atkinson SKC Brook F. Reproduction. In: Dierauf LA Gulland FMD, editors. CRC Handbook of Marine Mammal Medicine. Boca Raton, FL: CRC Press (2001). 208 p.

[51] Kiyota M Yamaguchi Y Nishikawa F Kohyama K Cytological changes in vaginal smear and epithelium associated with the reproductive cycle in northern fur seal, *Callorhinus ursinus*. Bull Nat Resour Inst Far Seas Fish. (1999) 36:17–25.

[52] Hendricks DM Biochemistry and physiology of the gonadal hormones. In: Cupps PT, editor. Reproduction in Domestic Animals. San Diego, CA: Academic Press (1991). p. 81–118. 10.1016/B978-0-08-057109-6.50008-2

[53] McNeilly AS Martin RD Hodges JK Smuts GL. Blood concentrations of gonadotrophins, prolactin and gonadal steroids in males and in non-pregnant and pregnant female African elephants (*Loxodonta africana*). J Reprod Fert. (1983) 67:113–20. 10.1530/jrf.0.0670113 6401810

[54] Bowman BM Miller SC. Elevated progesterone during pseudopregnancy may prevent bone loss associated with low estrogen. J Bone Miner Res. (1996) 11:15–21. 10.1002/jbmr.5650110104 8770692

[55] Callard IP Bayne CG McConnell WF. Hormones and reproduction in the female lizard *Seeloporus eyanogenys*. Gen Comp Endocrinol. (1972) 18:175–95. 10.1016/0016-6480(72)90095-0 4550598

[56] Bourne AR Steward BJ Watson TG. Changes in blood progesterone concentration during pregnancy in the lizard *Tiliqua rugosa*. Comp Biochem Physiol. (1986) 84:581–4. 10.1016/0300-9629(86)90368-3 2874938

[57] Highfill DR Mead RA Sources and levels of progesterone during pregnancy in the viviparous garter snake, *Thamnophis elegans*. Gen Comp Endocrinol. (1975) 127:389–400. 10.1016/0016-6480(75)90206-3 1222903

[58] Hanks J Short RV. The formation and function of the corpus luteum in the African elephant, *Loxodonta Africana*. J Reprod Fert. (1972) 29:79–89. 10.1530/jrf.0.0290079 5017016

[59] Albrecht ED Townsley JD. Serum estradiol in mid and late gestation and estradiol/progesterone ratio in baboons near parturition. Biol Reprod. (1978) 18:247–50. 10.1095/biolreprod18.2.247 415766

[60] Walker LA Cornell L Dahl KD Czekala NM Dargen CM Joseph B Urinary concentrations of ovarian steroid hormone metabolites and bioactive follicle-stimulating hormone in killer whales (*Orcinus orca*) during ovarian cycles and pregnancy. Biol Reprod. (1988) 39:1013–20. 10.1095/biolreprod39.5.1013 3146355

[61] Steinman KJ Robeck TR O'Brien JK. Characterization of estrogens, testosterone, and cortisol in normal bottlenose dolphin (*Tursiops truncatus*) pregnancy. Gen Comp Endocrinol. (2016) 226:102–12. 10.1016/j.ygcen.2015.12.019 26718081

[62] Hirayama H Sawai K Moriyasu S Hirayama M Goto Y Kaneko E. Excess estrogen sulfoconjugation as the possible cause for a poor sign of parturition in pregnant cows carrying somatic cell clone fetuses. Reproduction. (2008) 136:639–47. 10.1530/REP-08-0157 18663016

[63] Ellinwood RM Brenner DL Hess JA. Testosterone synthesis in Rhesus fetal testes: comparison between middle and late gestation. Biol Reprod. (1980) 22:955–63. 10.1095/biolreprod22.4.955 6249409

[64] Nahid L Sirous K The effects of fetal gender on serum human chorionic gonadotropin and testosterone in normotensive and preeclamptic pregnancies. J Pregnancy. (2012) 2012:874290 10.1155/2012/874290 22518314 PMC3306902

[65] Duer C Carden M Schmitt D Tomasi T Utility of maternal serum total testosterone analysis for fetal gender determination in Asian elephants (*Elephas maximus*). Anim Reprod Sci. (2002) 23:47–52. 10.1016/S0378-4320(01)00147-6 11755716

[66] Altman J Lynch JW Nguyen N Alberts SC Gesquiere LR Life-history correlates of steroid concentrations in wild peripartum baboons. Am J Primatol. (2004) 64:95–106. 10.1002/ajp.20064 15356861

[67] Drea CM Endocrine correlates of pregnancy in the ring-tailed lemur (*Lemur catta*): implications for the masculinization of daughters Horm *Behav*. (2011) 59:417–27. 10.1016/j.yhbeh.2010.09.011 20932838

[68] Nabi G Aziz T Amin M Khan AA Effect of fetal sex on total levels of maternal serum testosterone. J Biol Life Sci. (2014) 5:58–63. 10.5296/jbls.v5i2.5228

[69] French JA Smith AS Birnie AK Maternal gestational androgen levels in female marmosets (*Callithrix geoffroyi*) vary across trimesters but do not vary with the sex ratio of litters. Gen Comp Endocrinol. (2010) 165:309–14. 10.1016/j.ygcen.2009.07.015 19646445 PMC2784116

[70] Houstmuller EJ de Jong FH Rowland DL Slob AK Plasma testosterone in fetal rats and their mothers on Day 19 of gestation. Physiol Behav. (1995) 57:495–9. 10.1016/0031-9384(94)00291-C 7753887

